# Nutritional Implications of Methotrexate in Osteoarthritis: A Systematic Review and Meta-Analysis

**DOI:** 10.2174/0115748871406171251115072808

**Published:** 2026-03-16

**Authors:** Saud Nayef Salem Aldanyowi, Abdulsalam Mohammed Aleid, Yousef Khalaf A Alghamdi, Hasan Ali Abdullah AlAidarous, Zainab Mohammed Aleid

**Affiliations:** 1 Department of Surgery, Medical College, King Faisal University, Hofuf, Ahsa, 31982, Saudi Arabia;; 2 Faculty of Medicine, Surgery, Albaha University, Al Baha, Saudi Arabia;; 3 Faculty of Medicine, Surgery Department, Albaha University, Al Baha, Saudi Arabia

**Keywords:** Disease-modifying agents, joint pain, methotrexate, osteoarthritis, rheumatoid, cochrane

## Abstract

**Introduction:**

Osteoarthritis (OA) is a prevalent degenerative joint condition with limited treatment options targeting disease modification. Methotrexate (MTX), widely used in rheumatoid arthritis for its anti-inflammatory properties, may offer similar benefits in OA. However, its effectiveness and nutritional implications in OA remain uncertain.

**Methods:**

We conducted a systematic review and meta-analysis following PRISMA guidelines, registered in PROSPERO (CRD42024592121). We searched PubMed, Web of Science, OVID, and Cochrane Central up to August 2024 for randomized controlled trials comparing MTX with placebo in OA patients. Risk of bias was assessed using the Cochrane RoB 2 tool, and data were synthesized using Review Manager (RevMan) 5.4.

**Results:**

Four trials involving 416 participants were included. MTX significantly reduced pain at 3 months (SMD = -0.33, *p* = 0.006) and 6 months (SMD = -0.53, *p* = 0.0004), and stiffness at 6 months (SMD = -0.48, *p* < 0.0001). No significant improvement in physical function was found in the primary analysis (SMD = -1.07, *p* = 0.09), but sensitivity analysis excluding one high-risk study showed a modest benefit (SMD = -0.34, *p* = 0.01). Adverse events were consistent with low-dose MTX use. No study reported folate levels, but folate supplementation is recommended to mitigate MTX-related nutritional risks.

**Discussion:**

MTX appears to offer symptom relief in OA and may enhance function in select patients. Nutritional monitoring, particularly folate status, should accompany MTX therapy.

**Conclusion:**

MTX shows promise as an adjunct therapy for OA. Larger, long-term trials incorporating nutritional parameters are warranted.

## 1 INTRODUCTION

 Osteoarthritis (OA) is characterized by the deterioration of joint cartilage and the subsequent disintegration of numerous important joint components. Mechanical stress that surpasses the joints’ ability to self-repair is the primary element thought to cause OA [1]. OA is the leading cause of chronic pain and disability worldwide [2]. As people age, they are more likely to develop OA, with knee OA as the most common form. Predictions for 2010 indicated that 3.8% of the global population would experience symptomatic knee OA [3]. Around the age of 50, the prevalence was found to be highest. In knee OA, the degree of discomfort is associated with local inflammation within the joints [4. Increased pain and structural abnormalities, including cartilage defects, bone marrow lesions, and the onset of OA in the knee, are associated with synovial effusion and synovitis within the joint space [5, 6]. Reducing inflammation can help modify effusion-synovitis, which in turn may slow down unfavorable structural changes and improve outcomes in OA.

Currently, there is no medical intervention that can slow down the deterioration caused by OA. Decreasing inflammation is a successful strategy for managing OA [7]. The use of nonsteroidal anti-inflammatory drugs is the mainstay of nonsurgical OA treatment [8].

Methotrexate is a folate antagonist that inhibits dihydrofolate reductase, which lowers the amount of DNA that is synthesized and the number of cells that replicate, particularly in cells that divide fast [9]. A number of autoimmune disorders, including rheumatoid arthritis, psoriasis, and certain malignancies, are commonly treated with this substance [10, 11]. Since methotrexate competitively blocks the recycling of dihydrofolate to tetrahydrofolate, it can deplete cellular folate pools, leading to macrocytic anaemia, stomatitis, and hepatotoxicity unless exogenous folic or folinic acid is provided [12, 13]. Current rheumatology and oncology guidelines therefore recommend routine folic-acid supplementation (*e.g*., 1-5 mg orally the day after each weekly methotrexate dose) and regular monitoring of serum folate and liver enzymes [14, 15]. Older adults with OA may already have sub-optimal dietary folate intake, making nutrition counselling and coordinated dietitian care particularly relevant when methotrexate is prescribed.

Methotrexate is being investigated for its ability to reduce inflammation and pain in patients with OA. This is accomplished by changing immune responses and lowering levels of proinflammatory cytokines. Even though its specific role and long-term advantages in this context are still being investigated, preliminary studies have suggested that methotrexate may relieve symptoms and decrease the progression of the illness in OA. This systematic review and meta-analysis was conducted to evaluate and assess the current evidence regarding the efficacy of methotrexate for OA. However, these potential therapeutic benefits must be weighed against its nutrition-related risks, underscoring the importance of integrating dietary folate optimisation into any methotrexate-based management plan for OA.

In other words, this systematic review and meta-analysis aims to evaluate the efficacy of methotrexate for OA while also synthesising evidence on its nutritional implications, particularly folate supplementation requirements, to provide a more holistic perspective for clinicians, dietitians, and researchers.

## 2 METHODS

We conducted this systematic review and meta-analysis in accordance with the Preferred Reporting Items for Systematic Reviews and Meta-Analyses (PRISMA) guidelines [12] and the instructions of the Cochrane Handbook for Systematic Reviews of Interventions [13]. This study was registered with PROSPERO (CRD42024592121).

### 2.1 Database Searching

A comprehensive review of the scientific literature was carried out by utilizing four prominent databases (PubMed, Cochrane Central Register of Controlled Trials, Web of Science, and Scopus) to locate published publications using a specific search strategy: ((Degenerative arthritis) OR osteoarthritis) AND (methotrexate OR Otrexup OR Rasuvo OR RediTrex OR Trexall OR Xatmep), until August 2024 with minor modifications to accommodate the specific requirements of each database. Our search considered only research articles published in English. Non-English articles were excluded due to limited access to reliable translation resources and the potential risk of misinterpretation of clinical data. We acknowledge that this introduces a possible language bias, which may affect the comprehensiveness of our findings.

### 2.2 Inclusion and Exclusion Criteria

#### 2.2.1 Inclusion Criteria

We included all randomized clinical trials (RCTs), specifically those with the following PICO (P: patients with OA; I: methotrexate; C: placebo; O: focusing on posttreatment pain and functional performance).

#### 2.2.2 Exclusion Criteria

The meta-analysis excluded articles that did not fit the requirements of RCTs and clinical comparative studies. Exclusion criteria included reviews, letters to the editor, abstracts, comments, non-human investigations, and trials that compare the use of methotrexate to treatments other than placebo. Trials published in languages other than English were also excluded.

### 2.3 Study Selection and Data Extraction

To eliminate duplicates, the search results were imported into the EndNote software version 20 [14]. Afterwards, two reviewers assessed the titles and abstracts of the remaining papers using the Rayyan tool [15] to determine their suitability for the study. If necessary, a third reviewer would intervene after the original reviewers discussed and attempted to resolve any discrepancies in their ratings. Selected articles underwent a comprehensive evaluation process. Furthermore, we meticulously reviewed the citations used in the chosen publications to ensure a thorough exploration of potential directions for future research. In the end, two impartial evaluators manually collected the data and recorded the results on a standardized Google page. This document provides comprehensive information, including the year of publication, target audience, initial attributes, study sites, and baseline characteristics.

### 2.4 Efficacy Outcomes

#### 2.4.1 Primary Outcomes

Primary outcomes of this study included pain scores at 3 months and 6 months posttreatment.

#### 2.4.2 Secondary Outcomes

The secondary outcomes of this study primarily focused on the patients’ performance status, including stiffness and physical functional scores. While nutritional data were not primary endpoints of the included studies, we reviewed all included trials for any reported adverse events linked to folate deficiency or nutritional disturbances associated with methotrexate use. Unfortunately, none of the included studies reported data on folate levels, dietary intake, or related adverse effects. This highlights a gap in current literature and warrants attention in future OA-related trials involving methotrexate.

### 2.5 Risk-of-bias Assessment

To assess the risk of bias in the clinical trials that were included, we employed the revised Cochrane risk-of-bias tool for randomized trials (RoB 2) [16]. The tool is organized based on separate domains of bias, each addressing different aspects of trial design, implementation, and documentation. Each domain uses a distinct set of “signaling questions” to gather information about trial characteristics that are pertinent to the potential for bias. An algorithm processes the responses to these questions to provide a recommended assessment of the risk of bias in each domain. This assessment can be categorized as either “low” or “high” risk of bias, or it can indicate “some concerns.” Two separate reviewers individually scrutinized each domain. The purpose of this assessment was to gain a comprehensive understanding of the risk of biases that could affect the accuracy and consistency of the included studies.

### 2.6 Statistical Analysis

Meta-analysis was performed using Review Manager software (RevMan v.5.4) [17] on the extracted outcome data present in at least three of the included studies. Regarding continuous outcomes, data were pooled as standardized mean differences (SMDs) with 95% confidence intervals (CIs) due to differences in outcome reporting scales. The level of statistical significance was set to be p < 0.05. If means and standard deviations were not provided, we calculated them from other statistical indices. The change from baseline values was extracted instead of final values. In case of the absence of the SD of change, final values were extracted. Data represented by figures were extracted using the Plot digitizer online app [18]. A random-effects model (inverse variance) was adopted rather than a fixed-effects model, yielding a more conservative estimate of the pooled effect and more generalizable results. Heterogeneity was assessed by the chi-square test with a probability value of *p* ≤ 0.1 and an I² value >40%. We are concerned more with the I² value as I^2^ test performs better than the chi-square test when the number of included studies is small. Finally, in cases of significant heterogeneity, we conducted a sensitivity analysis of the data.

#### 2.6.1 Subgroup Analysis

Subgroup analysis based on joint type (*e.g*., knee *vs*. hand OA) and methotrexate dose was considered. However, due to the small number of eligible studies (n=4) and limited stratified data, such analyses were not feasible. We recommend that future RCTs report outcomes stratified by joint involvement to support more robust subgroup analysis.

#### 2.6.2 Sensitivity Analysis

Sensitivity analysis was conducted when significant heterogeneity was observed, to assess the stability of pooled estimates.

## 3 RESULTS

### 3.1 Study Selection

After conducting a comprehensive analysis of the existing literature, we identified 602 relevant records across various databases, including PubMed, Cochrane Central, OVID, and Web of Science. EndNote was used to optimize the data entry process, resulting in a final count of 453 records after eliminating 149 duplicates. After carefully examining the titles and abstracts, we chose 15 articles that met our study criteria. After carefully reviewing the entire text, we eliminated seven records that did not meet specific criteria, leaving us with a final selection of four studies (Kingsbury 2024 [19], Wang 2023 [20], Moghaddam 2019 [21], and Ferrero 2021 [22]) that were included for qualitative synthesis and meta-analysis. Furthermore, the reference lists of these research studies were thoroughly scrutinized to identify any relevant publications that might have been missed initially. Fig. (**1**) (PRISMA flow diagram) has been updated with clearer labels and footnotes that specify the main exclusion reasons (including language and non-RCT design).

### 3.2 Characteristics of the Studies

We identified four trials involving 416 participants conducted between 2019 and 2024. Kingsbury (2024) and Moghaddam (2019) studied knee OA, whereas Wang (2023) and Ferrero (2021) examined hand OA. All were prospective, parallel-group RCTs comparing various methotrexate regimens with placebo. Table **1** has been relabelled “Study Design Summary”, and Table **2** has been expanded to include any reported nutritional baseline characteristics (body-mass index, alcohol use, folate supplementation, serum folate/vitamin B12). No trial reported baseline serum folate or routine folate supplementation at enrolment; only mean BMI values were available.

### 3.3 Risk of Bias and Quality Assessment

The risk-of-bias assessment within the included RCTs was conducted using the RoB 2 tool. The risk-of-bias assessment revealed that three clinical trials (Kingsbury 2024, Wang 2023, and Ferrero 2021) were deemed as low risk of bias and high quality of evidence. However, one clinical trial (Moghaddam 2019) showed a high risk of bias. Fig. (**2**) presents a detailed risk-of-bias graph about the judgment of each domain in all included trials.

### 3.4 Efficacy Outcomes

#### 3.4.1 Primary Outcomes

##### 3.4.1.1 Pain Score after 3 Months

Pain scores were measured in three clinical trials using different assessment methods. Therefore, we adopted SMD for analysis. A meta-analysis was conducted, and results of pooled treatment effect showed statistically significant difference between methotrexate and placebo, favoring the methotrexate group, which was associated with less pain score (SMD = -0.33, 95% CI [-0.56, -0.^10^], *p* = 0.006) and with no heterogeneity detected (Heterogeneity: Tau² = 0.00; Chi² = 0.50, df = 2 (*p* = 0.78); I² = 0%) as shown in Fig. (**3**).

##### 3.4.1.2 Pain Score after 6 Months

The meta-analysis results showed a statistically significant lower pain score in the methotrexate group compared to the placebo (SMD = -0.53, 95% CI [-0.82, -0.^24^], *p* = 0.0004) with no significant heterogeneity detected (Heterogeneity: Tau² = 0.03; Chi² = 3.35, df = 2 (*p* = 0.19); I² = 40%) as illustrated in Fig. (**4**).

#### 3.4.2 Secondary Outcomes

##### 3.4.2.1 Stiffness Score after 6 Months

A meta-analysis was conducted, and the results showed a statistically significant less stiffness score, favoring methotrexate over placebo (SMD = -0.48, 95% CI [-0.70, -0.26], *p* < 0.0001) with no heterogeneity (Heterogeneity: Tau² = 0.00; Chi² = 0.72, df = 2 (*p* = 0.70); I² = 0%) as shown in Fig. (**5**).

##### 3.4.2.2 Physical Function Score after 6 Months

The meta-analysis results for the physical function score showed no statistically significant difference between methotrexate and placebo (SMD = -1.07, 95% CI [0.^17^], *p* = 0.09) with substantial heterogeneity detected (Heterogeneity: Tau² = 1.16; Chi² = 51.42, df = 2 (*p* < 0.00001); I² = 96%) that could be solved by conducting sensitivity analysis. The sensitivity analysis results showed that by excluding Moghaddam 2019, heterogeneity was resolved (*p* = 0.57, I^2^= 0%), and the treatment effect became statistically significant, favoring methotrexate (SMD = -0.34, 95% CI [-0.60, -0.0^8^], *p* = 0.01). Sensitivity analysis that excluded the high-bias study (Moghaddam 2019) eliminated heterogeneity (*I²* = 0 %) and yielded a significant benefit (SMD = –0.34; *p* = 0.01), Fig. (**6**).

### 3.5 Nutritional Adverse Events and Safety

None of the included RCTs prospectively measured serum folate, vitamin B12, homocysteine concentrations, or dietary intake. No study reported macrocytic anaemia, mucositis, or laboratory evidence of folate depletion. Mild gastrointestinal complaints (nausea or dyspepsia) were documented in two trials but occurred with similar frequency in placebo groups. No hepatotoxic events were attributed to methotrexate. Consequently, a quantitative synthesis of nutrition-related adverse events was not feasible, and the evidence base for long-term nutritional safety remains insufficient.

## 4 DISCUSSION

Osteoarthritis is a degenerative joint disease characterized by the breakdown of cartilage, leading to pain, stiffness, and reduced joint function. Methotrexate is proposed to treat OA by reducing inflammation and modulating immune responses, potentially slowing the progression of joint damage.

### 4.1 Main Findings

This systematic review and meta-analysis compared the effectiveness of methotrexate *versus* placebo in patients suffering from OA, in terms of pain relief and function improvement. The analysis identified several issues that make a case for methotrexate in the management of OA.

The most important outcome evaluated was the decrease in pain score after three and six months of treatment. In the three studies conducted, it was demonstrated that methotrexate significantly reduces pain compared to a placebo. Upon the completion of the second month of treatment, the patients receiving methotrexate reported greater-than-expected levels of pain reduction, indicating its effectiveness in minimizing pain in the patients very early in the course of treatment. This relief was preserved and even increased further at the sixth month, thus supporting the use of methotrexate in pain relief among patients with OA over a longer duration.

The meta-analysis also assessed secondary outcomes, including stiffness and physical function. Secondary outcomes included reduction of stiffness, a significant challenge among patients diagnosed with OA. The analysis showed that stiffness improved more in those taking methotrexate compared to placebo. These results are crucial since stiffness affects many aspects of the patients' day-to-day activities.

However, the results for physical function were more complex. Firstly, the meta-analysis did not detect any difference between the methotrexate and placebo groups, and there was considerable heterogeneity across the studies. This observed variability meant that the results were likely confounded by other intervening variables, such as study designs and patient types, among others. When this was further investigated using sensitivity analysis, it was demonstrated that excluding one article resolved the heterogeneity, and an increase in physical functioning was observed in patients taking methotrexate. This suggests that methotrexate may also help improve physical activities for patients with OA, although this effect appears to be more specific and might depend on certain patient profiles or study settings.

In conclusion, the findings of this systematic review and meta-analysis support the use of methotrexate in the treatment of OA as a measure to alleviate pain and stiffness in patients, and also to improve physical activity levels. The responses from patients with OA are encouraging, especially considering the limited management options available, and these results highlight the need for further studies to uncover all the effects of methotrexate when treating this disease [23].

### 4.2 Nutritional Implications

Methotrexate is an anti-folate agent that competitively inhibits dihydrofolate reductase, depleting intracellular folate pools and predisposing patients to macrocytic anaemia, stomatitis, and hepatotoxicity unless exogenous folic or folinic acid is provided. In rheumatoid arthritis, weekly folic-acid supplementation (1-5 mg 24 h after each methotrexate dose) is standard of care; similar protocols should be adopted for OA patients [24].

Older adults with OA often have sub-optimal dietary folate, B-vitamin, or protein intake, and many use alcohol or non-steroidal anti-inflammatory drugs that further stress hepatic pathways. Dietitians should therefore:

1. Counsel on folate-rich foods (dark-green vegetables, pulses, fortified grains) and prescribe pharmacological folate where required.
2. Monitor full blood count, liver enzymes, and, ideally, serum folate or homocysteine every 8-12 weeks during methotrexate therapy [25].
3. Address concomitant deficiencies (vitamin B12, iron) that can mimic or compound methotrexate-related cytopenias.

Future OA trials should prospectively capture nutritional status (BMI, dietary intake, serum folate/B12) and report nutrition-related adverse events to clarify long-term safety.

### 4.3 Clinical Implications

This systematic review as well as meta-analysis contains important information for managing OA, considering the limited treatment options available for this condition. It has been suggested that methotrexate, which is a drug traditionally used for inflammatory conditions, such as rheumatoid arthritis, should now also be considered as a treatment for OA, particularly for those patients suffering from continuous pain and stiffness. Given that methotrexate has been shown to alleviate pain and joint stiffness, it would be a useful augmentation strategy, particularly in patients with inadequate response to anti-inflammatory medications, nonsteroidal anti-inflammatory drugs, and physiotherapy. Moreover, the possible relative enhancement of physical function, as indicated by the sensitivity analysis, suggests that methotrexate may not only be effective in controlling symptoms in patients with OA but also improve their physical ability to perform activities of daily living, thereby enhancing their overall quality of life. The surgeon would administer methotrexate as part of a comprehensive management approach, particularly in instances where inflammation may be contributing to the worsening of the clinical picture. Given the considerable interindividual variability in response to it, as well as the need to manage adverse events associated with long-term methotrexate use, proper patient selection and oversight are crucial in this instance. This assessment underscores the importance of carrying out additional clinical studies to optimize the scheme of the administration in terms of the most beneficial for these patients and the duration of the therapy and for which subgroups of patients with OA would be most effective.

## 5 STUDY LIMITATIONS

It is important to acknowledge certain limitations when interpreting the findings. Firstly, our results are based on only four RCTs, each with a small sample size. Therefore, additional high-quality RCTs with larger sample sizes are necessary to confirm these results in the future. The studies did not fully report their outcomes, so estimates were calculated using statistical methods. However, these estimates inevitably included a margin of error. Although there was no heterogeneity (I^2^ = 0), variations in the doses, number of administrations, durations, initiation times, standard treatment that the patients are taking, follow-up duration, and sample size could introduce biases and potentially impact the pooled outcomes. Consequently, more RCTs are needed to assess if the study characteristics have a potential impact on the effect of methotrexate. Despite the fact that the results for the 1-year follow-up are available, we did not analyze them for the pooled analysis from two studies, as we have some concerns. In this review, we analyzed patients assessed during the first 6 months only. Consequently, studies with longer follow-up periods are required to determine whether the effectiveness of methotrexate treatment remains consistent or improves further over time. Given the chronic nature of osteoarthritis (OA), it is crucial to assess the sustained efficacy of the treatment beyond one year. Finally, conducting a subgroup analysis based on the specific joints affected and evaluating potential publication bias was not possible with the currently included studies.

## CONCLUSION

The current systematic review as well as meta-analysis suggests that methotrexate may be a useful adjunct for patients with OA, showing statistically significant reductions in pain within 3 months that are maintained through 6 months and accompanied by meaningful improvements in stiffness. Evidence for enhanced physical function is modest and emerged only after sensitivity analysis, indicating that functional gains are heterogeneous and context-dependent.

Since methotrexate antagonises folate metabolism, its clinical adoption in OA must be coupled with routine folic-acid supplementation and ongoing nutritional monitoring (full blood count, liver enzymes, ± serum folate/B12). Such measures are standard in rheumatoid arthritis care and should be extended to OA populations-especially older adults who may already have sub-optimal dietary folate.

While these findings highlight methotrexate’s potential for OA patients who do not respond adequately to conventional therapies, the evidence base remains limited to four small RCTs with short follow-up. Larger, longer-term trials that incorporate nutritional outcomes and detailed safety monitoring are essential to corroborate these observations, optimise dosing regimens, and identify subgroups most likely to benefit.

## Figures and Tables

**Fig. (1):**
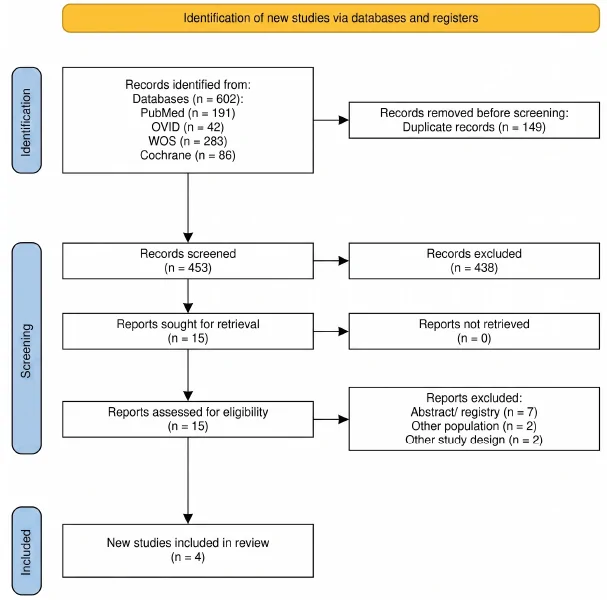
PRISMA flow diagram of the selection process.

**Fig. (2):**
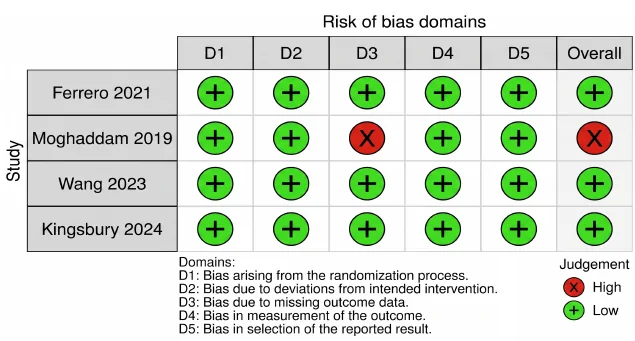
Risk-of-bias graph.

**Fig. (3):**
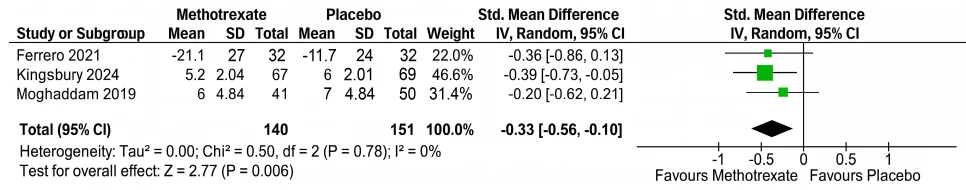
Forest plot of the pain score after 3 months. **Abbreviation:** SD, standard deviation.

**Fig. (4):**
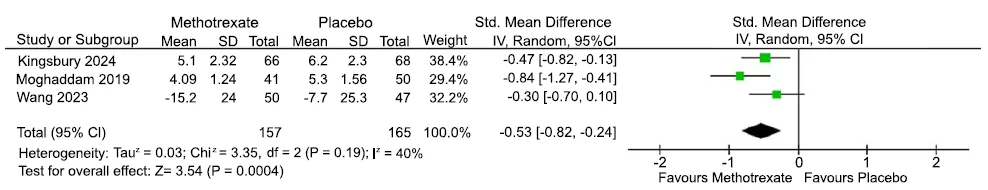
Forest plot of the pain score after 6 months. **Abbreviation:** SD, standard deviation.

**Fig. (5):**
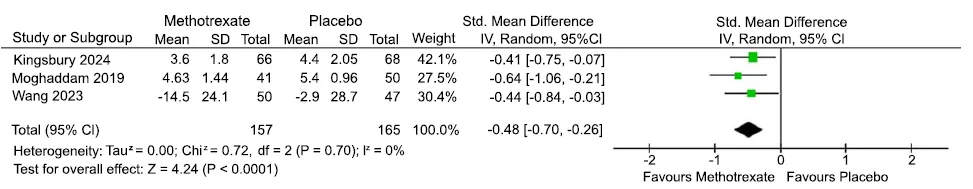
Forest plot of the stiffness score after 6 months. **Abbreviation:** SD, standard deviation.

**Fig. (6):**
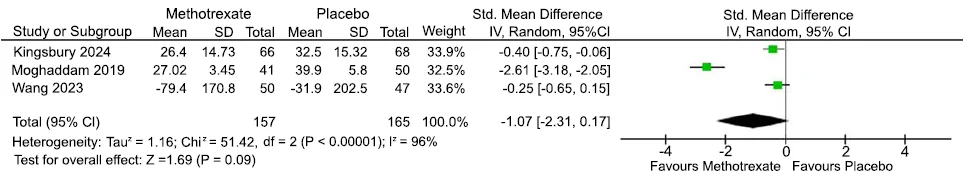
Forest plot of the physical function score after 6 months. **Abbreviation:** SD, standard deviation.

**Table 1:** Summary of the included studies.

**Study ID**	**Study Design**	**Location**	**Population**	**Intervention**	**Comparator**	**Total Sample Size**	**Sample Size**	
							**Intervention**	**Comparator**
Kingsbury (2024)	RCT	UK	Patients aged 18 years or older with a diagnosis of primary knee OA who meet the American College of Rheumatology clinical criteria	10 mg of methotrexate for 2 weeks, followed by 15 mg for 2 weeks, 20 mg for 2 weeks, and up to 25 mg for the remainder of the study	Placebo	155	77	78
Wang (2023)	RCT	Australia	Patients aged 40–75 years with hand joint pain on most days in the past 3 months, with a pain score of at least 40 mm on a 100-mm visual analogue scale in the past 7 days, and with hand osteoarthritis that meets the American College of Rheumatology criteria	Methotrexate 20 mg orally, once weekly, for 6 months	Placebo	97	50	47
Moghaddam (2019)	RCT	Iran	Patients aged 45–75 years with moderate to severe knee OA with noninflammatory joint effusion	7.5 mg of methotrexate weekly, then increase to 15 mg weekly after the first month	Placebo	100	50	50
Ferrero (2021)	RCT	France	Patients aged 45-85 years diagnosed with hand OA according to the American College of Rheumatology criteria, with at least one joint with erosion on radiography of both hands, and with visual analogue scale pain levels >40 mm for >3 months	10 mg methotrexate group (administered four methotrexate tablets on Mondays and one folic acid tablet weekly) for 12 months	Placebo	64	32	32

**Abbreviations:** OA, osteoarthritis; RCT, randomized clinical trial.

**Table 2:** Baseline characteristics of the patients of the included studies.

**Study ID**	**Study Arms**	**Sample Size**	**Age Mean (SD)**	**Sex (Female), n (%)**	**BMI, Mean (SD)**	**Symptoms Duration in Years, Mean (SD) Mean (SD)**	**Kellgren and Lawrence Grade, n (%)**	
							**Grade 3**	**Grade 4**
Kingsbury (2024)	Methotrexate	77	61.5(9.79)	50 (64.9)	31.9(6.25)	9.3(8.03)	22(28.6)	13(16.9)
	Placebo	78	60.4(9.59)	49 (62.8)	33.7(6.48)	9.3(8.33)	27(34.6)	16(20.5)
Wang (2023)	Methotrexate	50	61.4(6.1)	34 (68)	29.7(5)	7(5.8)	12(24)	13(27)
	Placebo	47	61.5(7.3)	34 (72)	27.9(5)	6.8(8.1)	13(28)	12(26)
Moghaddam (2019)	Methotrexate	41	53.75(6.59)	26(63.4)	NR	8.34(3.63)	21(51.2)	20(48.8)
	Placebo	50	52.42(7.19)	31(62)	NR	7.16(4.11)	31(62)	19(38)
Ferrero (2021)	Methotrexate	32	64.9(7)	31 (97)	24.2(4)	NR	NR	NR
	Placebo	32	67.5(8)	29 (91)	24.6(4)	NR	NR	NR

**Abbreviations:** BMI, body mass index; NR, not reported; SD, standard deviation.

## Data Availability

The data and supportive information are available within the article.

## LIST OF ABBREVIATIONS

BMI: Body Mass Index
CI: Confidence Interval
DNA: Deoxyribonucleic Acid
MTX: Methotrexate
NSAIDs: Nonsteroidal Anti-inflammatory Drugs
OA: Osteoarthritis
PRISMA: Preferred Reporting Items for Systematic Reviews and Meta-Analyses
PROSPERO: International Prospective Register of Systematic Reviews
RCT: Randomized Controlled Trial
RoB 2: Revised Cochrane Risk of Bias Tool for RCTs
SD: Standard Deviation
SMD: Standardized Mean Difference
VAS: Visual Analogue Scale

## STANDARDS OF REPORTING

PRISMA guidelines have been followed.

## SUPPLEMENTARY MATERIAL

PRISMA checklist is available on the publisher’s website along with the published article.
